# Determination of Brain Tissue Samples Storage Conditions for Reproducible Intraoperative Lipid Profiling

**DOI:** 10.3390/molecules27082587

**Published:** 2022-04-18

**Authors:** Stanislav I. Pekov, Evgeny S. Zhvansky, Vasily A. Eliferov, Anatoly A. Sorokin, Daniil G. Ivanov, Eugene N. Nikolaev, Igor A. Popov

**Affiliations:** 1 Skolkovo Institute of Science and Technology, 121205 Moscow, Russia; 2 Moscow Institute of Physics and Technology, 141701 Dolgoprudny, Russia; evgeny.zhvanskij@phystech.edu (E.S.Z.); v.eliferov@yandex.ru (V.A.E.); lptolik@gmail.com (A.A.S.); daniil.ivanov@phystech.edu (D.G.I.); 3 Siberian State Medical University, 634050 Tomsk, Russia; 4 Department of Biochemistry and Systems Biology, Faculty of Health and Life Sciences, Institute of Systems, Molecular and Integrative Biology, University of Liverpool, Liverpool L69 3BX, UK; 5 National Medical Research Center for Obstetrics, Gynecology and Perinatology Named after Academician V.I. Kulakov, 117997 Moscow, Russia

**Keywords:** molecular profiling, neurosurgery, ambient ionization mass spectrometry, cosine similarity

## Abstract

Ex-vivo molecular profiling has recently emerged as a promising method for intraoperative tissue identification, especially in neurosurgery. The short-term storage of resected samples at room temperature is proposed to have negligible influence on the lipid molecular profiles. However, a detailed investigation of short-term molecular profile stability is required to implement molecular profiling in a clinic. This study evaluates the effect of storage media, temperature, and washing solution to determine conditions that provide stable and reproducible molecular profiles, with the help of ambient ionization mass spectrometry using rat cerebral cortex as model brain tissue samples. Utilizing normal saline for sample storage and washing media shows a positive effect on the reproducibility of the spectra; however, the refrigeration shows a negligible effect on the spectral similarity. Thus, it was demonstrated that up to hour-long storage in normal saline, even at room temperature, ensures the acquisition of representative molecular profiles using ambient ionization mass spectrometry.

## 1. Introduction

The metabolism alterations that accompany malignancy allows to implement rapid molecular techniques in clinical practice [1,2,3,4]. A variety of ambient mass spectrometry methods are proposed to discriminate cancer tissues intraoperatively [5,6,7]. Real-time tissue analysis performed in vivo is undoubtedly time-effective and beneficial, but requires the arrangement of expensive, complex instruments in every operating room [8,9,10]. The online investigation of ex vivo tissue samples is much more cost- and labor-effective, as one laboratory is capable of supporting multiple surgeries in parallel [11,12,13,14,15]. Thus, regarding neurosurgery [16,17,18], the tumor excision time is substantially lower than the total time of the surgical intervention, and ex vivo molecular profiling is still faster than traditional histopathology or costly intraoperative neuroimaging [19].

The use of ambient mass spectrometry for ex-vivo tissue analysis in the clinical environment requires the implementation of tissue collection and transfer protocols as part of a routine biopsy investigation pipeline [19]. Applying specific solvents or conditions for short-term sample storage complicates the method’s integration, as additional instruments, consumables, and training are required for the personnel. On the other hand, the utilization of widespread liquids, such as distilled or deionized water, or normal saline, along with the absence of specific requirements for short-term storage, allows the easy implementation of mass spectrometry techniques in clinical practice, especially in the early stages of clinical trials.

The activity of phospholipases and other enzymes at room temperature may still persist [20], but in contrast to proteomics, it is not expected to have a crucial influence on lipid profiles [21]; however, the oxidation and degradation of lipids might persist after short exposition without refrigeration [22,23]. It was demonstrated [24] that the 1-h storage of brain tissues at room temperature does not significantly affect the lipid profile, but a detailed investigation of short-term lipid profile stability is required to implement molecular profiling in a clinic. In this work, we attempted to evaluate the effects of short-term storage in various conditions on the stability and reproducibility of brain tissue lipid profiles.

## 2. Results and Discussion

In this study, the cerebral cortexes of rats from the same litter grown in identical conditions since birth were chosen as the model tissue samples. Since the number of samples required to analyze the effects of storage conditions on the brain tissues’ molecular profiles is relatively big, we had to obtain samples from a total of three animals. All of these rats were grown in the vivarium as control animals for various studies. They were fed with a balanced diet, and only donated blood samples throughout their lives. However, even in this case, natural variability may occur in the brain lipid composition of each animal, so it should be specifically noted that the samples from each participating animal were included in consideration to minimize possible individual effects. The samples were stored in different conditions to evaluate the influences of the short-term storage and washing (essentially required to remove blood traces) media composition and temperature on the molecular profiles required for the development of simple sample collection, transportation from an operating room to the laboratory, and treatment before the analysis.

A multifactor experimental design (see Section 3.2 in Materials and Methods) was selected to evaluate the effects of the temperature and composition of the solution used for sample storage after resection and pretreatment. Supposing that the alternating factors will not interfere with each other, this design allows us to minimize the number of samples (i.e., sacrificed animals) required for the experiment. Therefore, the total number of samples used for the multifactor experiment was 72.

The intraoperative methods used for cancer tissue discrimination rely not on the specific biomarkers, but also on the complex analysis of the molecular profiles [25] of the analyzed tissue. In this study, we use the Inline Cartridge Extraction (ICE) [14] ambient ionization mass spectrometry technique to rapidly obtain molecular profiles of brain tissues. Similar to many other ambient ionization methods [5,6], it precludes lipid identification (see Figures S1 and S2 in Supplementary Materials) and relies on the analysis of the sample’s total molecular profile, including the lipid component [4], which is the predominant component of molecular profiles obtained by ambient ionization mass spectrometry [10,11]. Multiple techniques for intraoperative tumor tissue classification [9,10] are based on a multivariate statistical analysis of the tissue profile, rather than on the identification of the molecular nature of the detected ions. Thus, in this work, we are focusing on the reproducibility of molecular profiles as a whole, and on how pre-measurement manipulations influence those profiles. The Spectra Similarity Matrices [26] (SSMs) based on cosine dissimilarity metrics were proposed as a tool of spectra stability and reproducibility evaluation, and utilized to assess the effects of the storage time and conditions on the acquired spectra.

**Figure 1:**
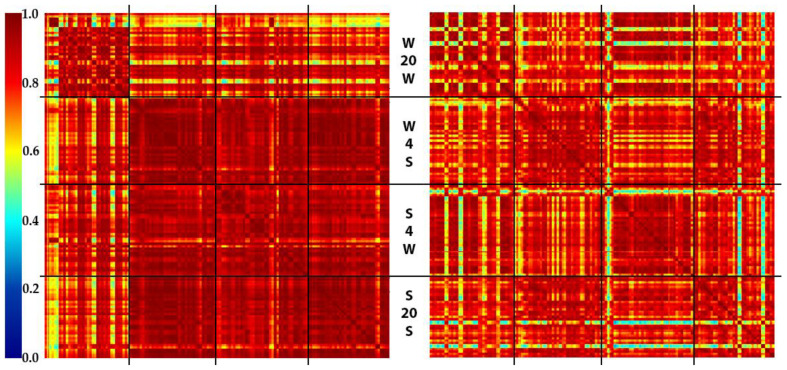
SSMs (normalized cosine dissimilarity) for the multifactor experiment (**left**—positive mode, **right**—negative mode), see Section 3.2 for details. The regions corresponding to saline-treated samples demonstrate higher spectra similarity, while the temperature does not seem to affect the molecular profiles. Black lines divide the SSMs into sections corresponding to identical conditions. From top to bottom: W20W—storage media, water; temperature, +20 °C; washing media, water. W4S—storage media, water; temperature, +4 °C; washing media, saline. S4W—storage media, saline; temperature, +4 °C; washing media, water. S20S—storage media, saline; temperature, +20 °C; washing media, saline.

The results have been visualized using SSMs and are presented in Figure 1. Each pixel of the SSM represents a cosine dissimilarity metric between two spectra indexed along the axes. For each sample, spectra are ordered by the acquisition time. The spectra of different samples are grouped by storage conditions. The resulting SSMs consist of four sections, which correspond to different storage and washing media and the storage temperature after the brain dissection. In each section of the matrix, the samples have been ordered in rows from top to bottom, and in columns from left to right, by the time of exposure at the corresponding temperature. The samples corresponding to identical conditions and times of exposure were mixed in a random order to prevent animal-specific correlations. Thus, each section of the SSM represents the similarity of the spectra obtained under similar conditions along the time course (one hour) of sample exposure [26,27]. The dark red pixels mark out spectra of high similarity. In contrast, the green or blue pixels show the instability of the detected molecular profiles in each measurement, or between the samples.

The high spectral variability in the regions corresponding to the water used for the sample storage (especially section W20W, both polarities) demonstrates substantial sample-to-sample variations. This reflects the effect of cell lysis caused by the osmotic shock during sample storage [23,28]. As expected, sample storage in normal saline prevents the degradation of the cells, providing more reproducible lipid profiles. The slight effect of lowered temperature maintenance are observed, resulting in the reduced heterogeneity of the corresponded regions on the SSM. However, this effect is not strong enough, and it can be ignored for the facilitation of routine procedures in a clinic.

The composition of the solution used for sample treatment before the analysis has lower effects compared to that of the storage solution. The SSM regions corresponding to water treatment are more stable, but detailed analyses of the spectra show that the total ion current appears to be lower. The reason for such behavior is also the cells lysis in the deionized water. Moreover, the samples treated with water demonstrate instable spectra for the first 1 to 3 min of measurement, so a delay occurred between cartridge mounting and spectra recording. No such instability was observed for samples treated with normal saline. The excess of normal saline may increase the rate of sodiated lipid adducts forming during ionization compared to water treatment; however, the unification of the sample transfer and treatment procedures will make this effect reproducible and tolerable during further mathematical analysis [7].

**Figure 2:**
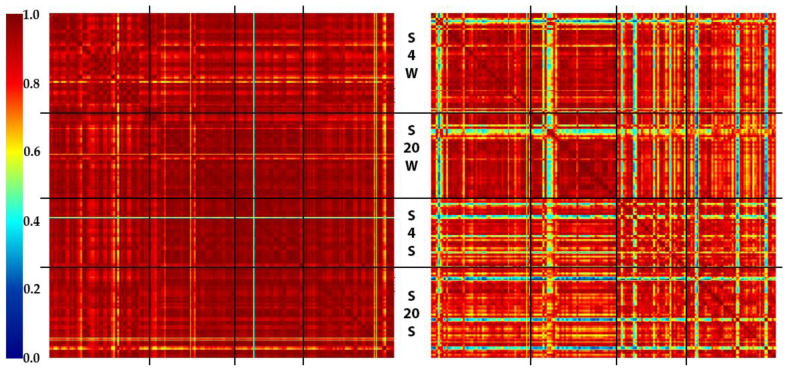
SSMs (normalized cosine dissimilarity) for the enhanced experiment (**left**—positive mode, **right**—negative mode), see Section 3.3 for details. The positive mode data do not show a difference between various storage conditions, while negative mode data are significantly more variable. Black lines divide the SSMs into sections corresponding to identical conditions. From top to bottom: S4W—storage media, saline; temperature, +4 °C; washing media, water. S20W—storage media, saline; temperature, +20 °C; washing media, water. S4S—storage media, saline; temperature, +4 °C; washing media, saline. S20S—storage media, saline; temperature, +20 °C; washing media, saline.

The additional set of samples (24 samples) was prepared and analyzed to prove the patterns revealed by the multifactor experiment. The results of the analysis of the enhanced set of data are visualized in Figure 2.

Again, the samples treated with water demonstrated higher reproducibility, but this appears to be a result of the lower intensity (and, thus, lower richness) of the spectra. The temperature’s effect on the reproducibility is still present (the mean cosine similarity between +4 °C and +20 °C regions is 0.961 positive mode; washing media—normal saline); however, this is comparable with the intraexperimental variability (the mean cosine similarity for +4 °C region is 0.962 and for the 20 °C region is 0.964; positive mode; washing media—normal saline).

**Figure 3:**
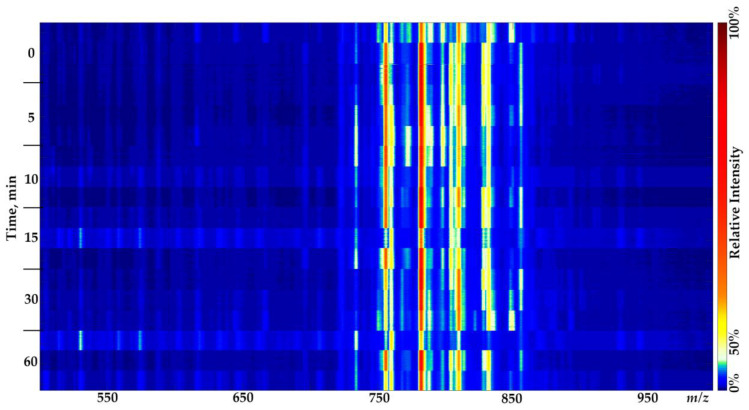
Ion map representing ICE molecular profiles obtained during the hour-long storage of rat brain tissue samples in normal saline at room temperature. Three samples from three animals per timestamp, positive mode. The intensity changes between measurements cause most of the visible alterations in spectra, while the overall molecular profile remains quite reproducible. The colormap has been adjusted for better visualization.

**Figure 4:**
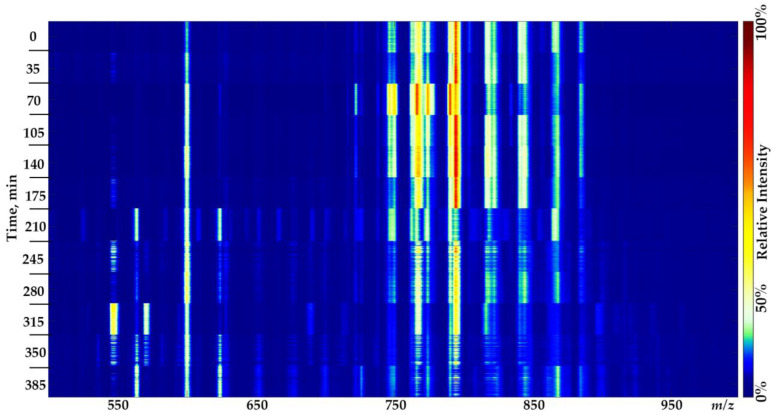
Ion map representing ICE molecular profiles obtained during six hours of rat brain tissue sample storage in normal saline at room temperature. One sample from one animal per timestamp, negative mode. Substantial alterations occur after 3 h of tissue storage. The colormap has been adjusted for better visualization.

**Figure 5:**
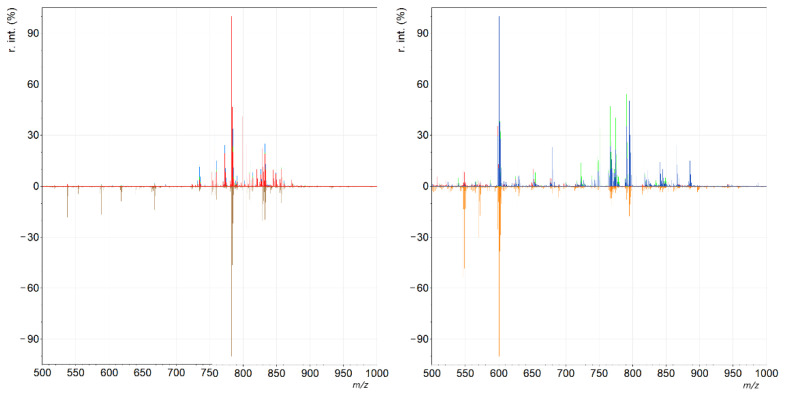
Spectra corresponding to samples analyzed after the incubation of samples in normal saline for 0 min (red), 35 min (green), 70 min (blue), and 315 min (brown). The three top spectra are almost identical, while 315 min of incubation at room temperature gave rise to signs of lipid degradation in the sample. Left: positive mode; right: negative mode. See Figures S3 and S4, and Tables S1 and S2 in Supplementary Materials for additional details.

As expected, no significant alteration in molecular profiles was observed within an hour of brain tissue storage in normal saline at room temperature [24] (the mean cosine similarity in positive mode is 0.985 and 0.963 in negative mode; washing media—normal saline). The dissimilar spectra observed for half of the samples, frozen immediately after dissection, are probably due to the fact that the blood residues were not entirely washed during the sample treatment. Therefore, an additional experiment was carried out to evaluate the effect of the long-term storage of resected tissues at room temperature. The obtained spectra show a negligible influence of hour-long storage in normal saline on lipid profiles (Figure 3, Figure 4 and Figure 5). Considerable changes in the lipid profiles (the mean cosine similarity in positive mode is 0.866 and 0.845 in negative mode; washing media—normal saline) occurred after only three hours of storage at room temperature, similar to what is shown elsewhere [29], related primarily to the loss of one of the fatty acid chains in lipids [24]. Although it is possible to propose some chemical annotations for unmodified lipid species in the molecular profiles of brain tissue samples (see Figures S1 and S2 in Supplementary Materials), the degradation products could not be identified within this study’s pipeline. The combination of unspecified hydrolysis and oxidation makes it impossible to annotate peaks in mass spectra using only the analysis of accurate masses and the isotopic distribution (see Tables S1 and S2 in Supplementary Materials).

## 3. Materials and Methods

### 3.1. Chemicals

Methanol (HPLC-grade), acetic acid (HPLC-grade) and sodium chloride (molecular biology grade) were obtained from Merck (Merck KGaA, Darmstadt, Germany).

### 3.2. Multifactor Experiment

Two male 18-month-old rats (Rattus norvegicus) from the same litter were humanely anesthetized and decapitated. The brain was immediately removed, bisected by longitudinal fissure, and the cerebral hemispheres detached from other brain structures. Twelve cerebral cortex samples (approximately 0.2–0.4 mm^3^ each) were immediately dissected from each hemisphere (a total of 96 samples, see Table S3 in Supplementary materials) and placed in a microtube with deionized water or normal saline at +4 °C or +20 °C. After the incubation of each dissected sample (from 0 to 60 min at the chosen temperature), it was frozen in liquid nitrogen and stored at −80 °C until analysis. From the four samples collected in identical conditions, only three were chosen randomly for the analysis. The sample measurement order was randomized. It was noted that no samples with identical collection conditions were measured on the same day.

### 3.3. Enhanced Experiment

The samples (a total of 36 samples, see Table S3 in Supplementary materials) from the third male animal from the same litter were obtained as described above; 24 samples were analyzed after randomization. Additionally, twelve samples were set aside, placed in normal saline at room temperature, and analyzed sequentially every 35 min (for 6 h total).

### 3.4. Mass Spectrometry

The freshly thawed samples were treated with deionized water or normal saline directly before the analysis. All samples were analyzed using the ICE technique and an LTQ Orbitrap XL ETD (ThermoFisher Scientific, San Jose, CA, USA) mass spectrometer as described elsewhere [14]. Briefly, the sample was placed inside a lab-made disposable cartridge (made from consumables provided by IDEX Health & Science LLC., Oak Harbor, WA, USA and GE Healthcare, Chicago, IL, USA) attached to the ESI interface of a mass spectrometer. The solvent flow (90% *v*/*v* methanol, 0.1% *v*/*v* acetic acid) extracts the analytes from the samples and delivers them into the ion source, while a glass microfiber filter inside the cartage protects the ESI needle from clogging. Mass spectra were acquired at the FTMS mass resolution set at 30,000 (FWHM at *m/z* 400 Da) for *m/z* values ranging from 500 to 1000 in the negative and positive modes, consecutively (30 s per polarity, 16 segments total). After the acquisition the ion source was washed with the solvent until the intensity of the signal decreased by at least 2 orders of magnitude from the level at the end of acquisition to avoid cross-contamination.

### 3.5. Data Analysis

Spectra similarity matrices with cosine measure were calculated as described elsewhere [26]. The width and step of the moving median filter were set to 5. The images were plotted using lab-made python and Matlab scripts.

## 4. Conclusions

The obtainment of a reliable molecular profile of resected tissue with ambient mass spectrometry requires specific sample handling procedures. First, cell lysis must be avoided, as it drastically decreases the stability, reproducibility, and intensity of mass spectra. The optimal way to reduce cell damage is to maintain the samples in normal saline at all times between dissection and mass spectrometry analysis. Plenty of sterile normal saline is always accessible in an operation room, so no specific efforts are required to keep samples intact. No significant difference in overall molecular profiles was found between samples stored at +4 °C and those stored at +20 °C for up to one hour, so room temperature is suitable for short-term storage during sample transfer between surgery and laboratory.

Lipid degradation in brain tissue samples is relatively slow, so we can see that the minor alterations in relative concentrations of various lipids caused by decomposition and oxidation do not affect the molecular profile noticeably, even after one hour of sample storage at room temperature. Moreover, significant changes in molecular profiles were experienced only after three hours of room temperature exposition in normal saline. Thus, the stability of lipid profiles after short-term exposition at ambient conditions facilitates the implementation of molecular profiling techniques in clinical practice. Placing tissue samples in normal saline without refrigeration provides a reliable way to preserve them before their analysis for up to one hour, so only the urgency of the response from the mass spectrometry laboratory limits the sample transfer time.

## Data Availability

The data related to this study are available from the corresponding author upon reasonable request.

## Supplementary Materials

The following supporting information can be downloaded at: https://www.mdpi.com/article/10.3390/molecules27082587/s1, Figures S1 and S2: Annotated mass spectra of rat brain tissue samples; Figures S3 and S4: Mass spectra correspond to samples analyzed after incubation of samples in normal saline for different periods of time; Table S1 and S2: Raw data for the experiments as specified in the main text; Table S3: The details on the sample preparation procedures.
