# Supplementary material for: Determination of Brain Tissue Samples Storage Conditions for Reproducible Intraoperative Lipid Profiling
Source: Molecules. 2022 Apr 18;27(8):2587. doi: 10.3390/molecules27082587 (PMC9029908; doi:10.3390/molecules27082587)
Supplement: Supplementary file 1 [file molecules-27-02587-s001.zip › molecules-1582474-supplementary.pdf]

Determination of brain tissue samples storage conditions for reproducible intraoperative lipid profiling

Stanislav I. Pekov, Evgeny S. Zhvansky, Vasily A. Eliferov, Anatoly A. Sorokin, Daniil G. Ivanov, Eugene N. Nikolaev, Igor A. Popov

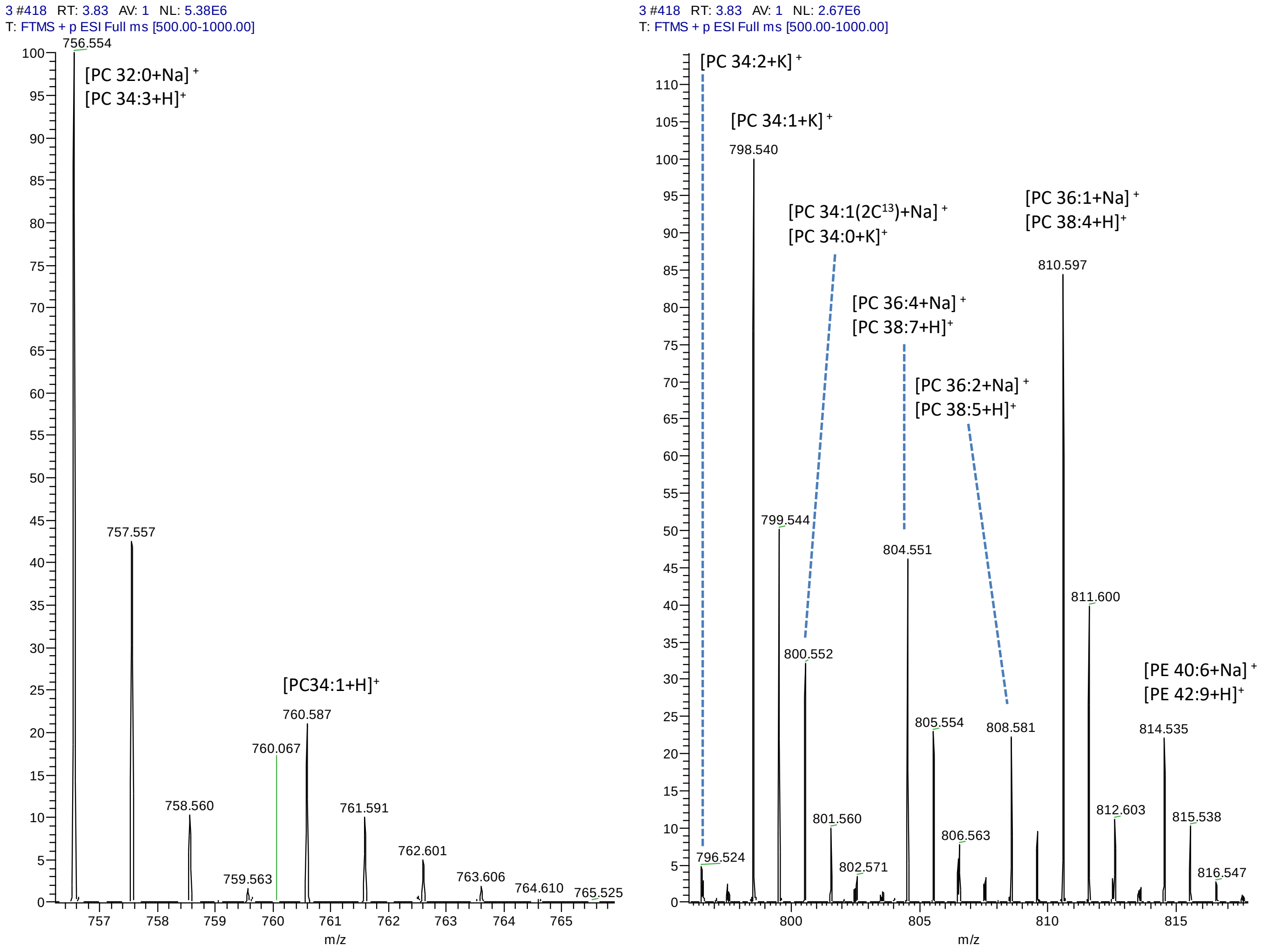

Figure S1. Mass spectra of rat brain tissue sample after 70 minutes incubation in normal saline at room temperature. Positive ion mode. The annotations for the monoisotopic peaks are based on accurate masses. The isotopic distribution is matched to the proposed lipid identifications. However, the presence of alkaline adducts precludes unambiguous identification.

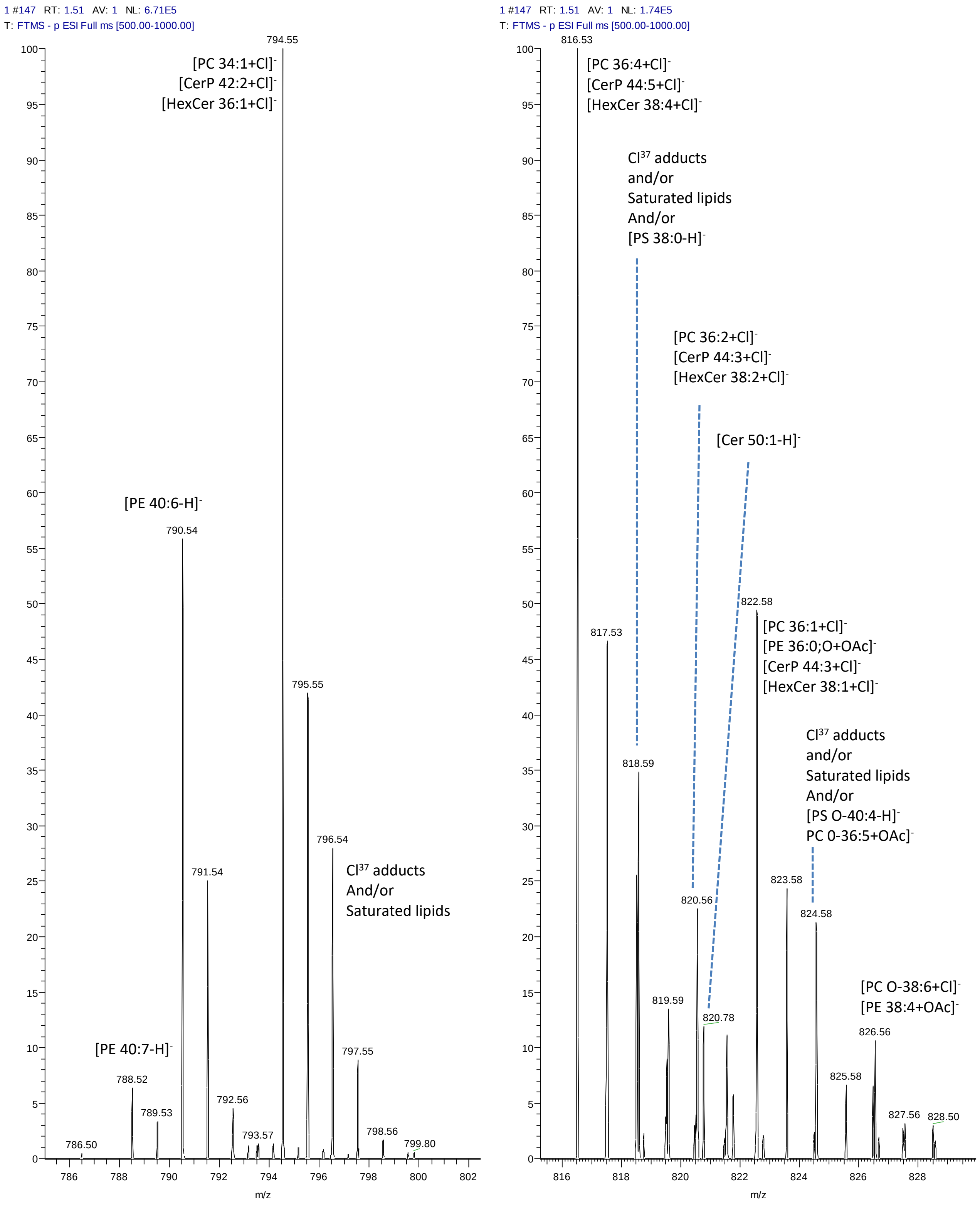

Figure S2. Mass spectra of rat brain tissue sample analyzed immediately after dissection. Negative ion mode. The annotations for the monoisotopic peaks are based on accurate masses. The isotopic distribution is matched to the proposed lipid identifications. However, the presence of various adducts precludes unambiguous identification.

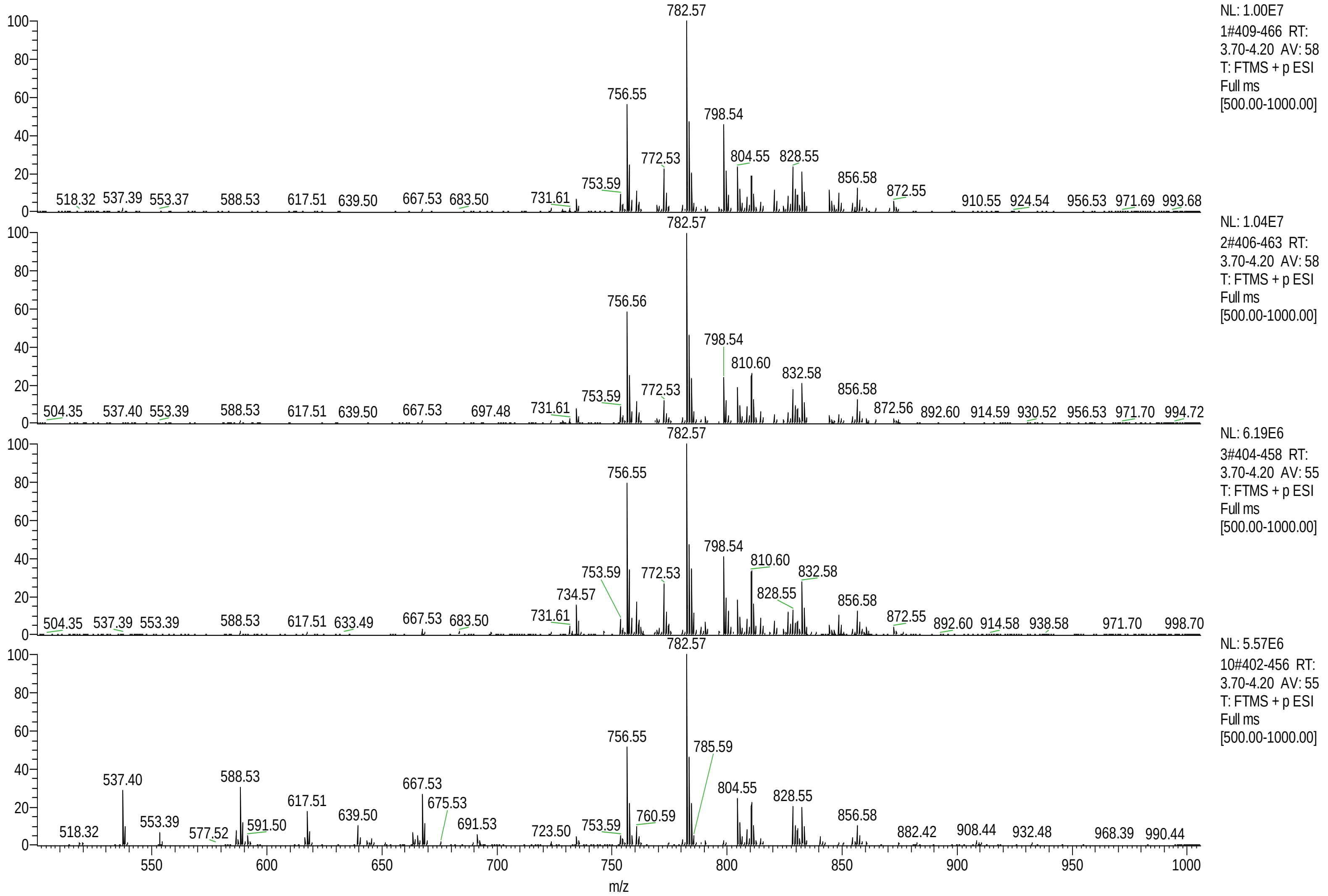

Figure S3. Mass spectra correspond to samples analyzed after incubation of samples in normal saline for 0 min, 35 minutes, 70 minutes, and 315 minutes (from top to bottom) at room temperature. Positive mode.

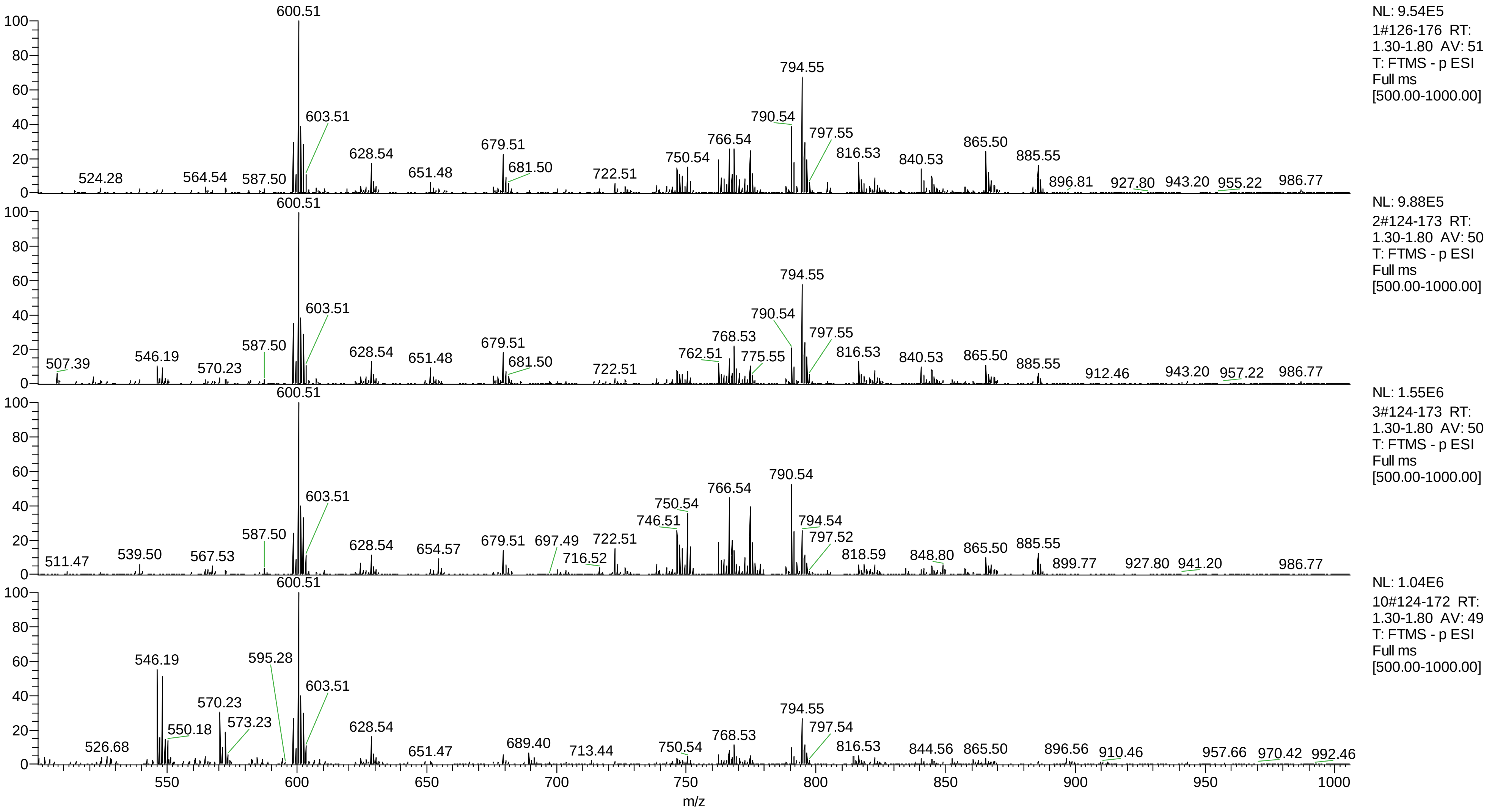

Figure S4. Mass spectra correspond to samples analyzed after incubation of samples in normal saline for 0 min, 35 minutes, 70 minutes, and 315 minutes (from top to bottom) at room temperature. Negative mode.

Table S1. Absolute intensities (counts) of the most intense ions (relative intensities above 10%) for samples analyzed after incubation of samples in normal saline. Positive mode.

Mass	0 min	35 min	70 min	315 min
537.39	194139,1			1613739
538.40				541146
553.37	103339,5			
553.39				360669,8
554.39				105997,8
586.52				432173,8
587.52				157060,7
588.53		110743,3	112306,9	1705294
589.54				665425,7
590.54				107767,4
591.50				267047,2
616.56				218722,2
617.51				995620,2
618.51				405613,8
639.49				563669,8
640.50				221047,3
643.53				143011,2
645.54				195905,7
663.50				360983,8
664.50				153039
665.51				292406,4
666.52				119320,2
667.53	147546,9	163969,1	169335,3	1474643
668.53				642489,5
669.53				132726,7
683.50	101759		104270,3	
691.53				309849,7
692.53				132827
697.48		102290,2		
723.49	165226,1	156887,9		102433,1
728.52	108560	112631,2		
731.61	188585,3	220916,9	268711,2	
732.61			112001,8	
734.57	661237,6	821808,1	982386,2	258758,2
735.57	281805,6	345575,8	437904,3	142767,1
746.51			100989,8	
751.57	103737,5			
753.59	906480,1	915987,8	492581,7	292983,1
754.54	374974,7	341179,8	171644,1	190870,5
754.59	396200,8	390634,2	214355,2	118710,7
755.54	158154,4	141078,4		

756.55	5670315	6063729	4921470	2879660
757.55	2471793	2622987	2120116	1226102
758.56	586865,4	649977,6	533850	288405,5
760.58	1102789	1193853	1061295	556500
761.59	487615,2	560984,7	483126,8	254409,5
762.60	125450,8	162803,7	258873,4	
768.59	104374,1	161357,6		
769.56	337526,9	225907,3	159525,1	
770.51	294389,4	218923,6	211134,7	
770.57	187370,4	137237,1	109596,5	
771.51	125533,4			
772.53	2260661	1209932	1658167	
773.53	988729,1	534961,9	731517,7	
774.54	318240,5	300576,9	343760,6	
775.55		120878,7	123461,3	
780.56	327965,3	308254,4	141803,4	172549
781.56	149251,2	137667,4		
782.57	10031188	10379485	6192565	5570574
783.57	4748582	4810066	2939630	2592360
784.58	2044158	2431479	2137080	1220756
785.59	461620,1	613890,2	711373	284623,2
786.51	213389	217737,7	137305	
786.59			124076,4	
787.51		100164,5		
788.62	133139	204571,3	232541,3	
789.62			110387,1	
790.54	289878	348945,8	411172,9	126741,8
791.54	136682,1	158176	191597,5	
796.52	231423,8	160946,4	123916,4	
796.58	100538,2	122583,5		
797.53	110533,7			
798.54	4598200	2525240	2524584	139135
799.54	2157744	1239880	1192800	
800.55	856473,6	438812,1	768803,2	
801.56	167690,2	143672,2	240341,4	
804.55	2344412	1958072	1131496	1365281
805.55	1189750	959685,6	563377,7	661840
806.51	129991,2		150322,9	
806.56	442480,2	378657,1	204184,6	238724,9
807.57	177569,2	154469,6		
808.58	749146	880020,8	524167,8	444157,5
809.58	348969,1	386094,2	241454,4	187713,6
810.60	1955093	2742696	2096127	1240616
811.60	911425	1311966	1010915	584374,6
812.60	224113,9	319891,4	264929,1	134906,5
814.54	525637,4	640950,8	544196,1	183540,4

815.54	269185,6	318994,8	270862,9	
820.52	1141064	448476,8	439795,8	
821.53	545652,1	213547,8	205014,4	
822.53	151269,4			
824.55	309787,5	179965,3	169214,4	
825.56	150409,3			
826.56	838598,6	566090,2	745580,8	
827.57	377513,5	266278,8	343735,3	
828.55	2344843	1863798	789982,7	1134586
829.55	1168751	978259,6	390361,1	587942
830.50	255195,6	137793,8	217936	
830.56	934901,5	806785,3	442963,9	488618,6
831.51	126579,4		106349,6	
831.57	359927,9	325009,1	195748,7	193256,5
832.58	2110535	2174888	1726140	1114555
833.59	1022612	1115705	871831,5	547523,6
834.59	275975,8	295521,1	251761,7	129412,6
840.53				252390,7
841.53				115336,4
844.52	1158369	436790,2	300797,8	
845.53	563606,5	197747,8	142878,4	
846.53	352441,8	130846,6	138973,6	
847.54	145714			
848.54	956052,9	464093,5	623552,4	
848.66		137653,6		
849.56	461769,4	231304,4	303147,1	
850.57	147576,2			
850.67		117698,6		
854.56	472603,6	380821,1	175719,7	224665,4
855.57	239722,6	191133,6		110908,8
856.58	1245971	1280513	779973,9	577902,2
857.58	617752	658085,3	395470,8	274440,8
858.59	225522,9	264447,6	196448,7	113336,2
860.61	179493,6	236593,8	244210,6	105676,2
861.62		116227	123973,4	
864.57	205401	216002,3		
865.57		102539,5		
870.54	202043,6			
871.55	102145,9			
872.55	533132,6	264750,5	261993,9	
873.56	261825,6	131249,8	131059,4	
874.54	152444,6	179477,1		

Table S2. Absolute intensities (counts) of the most intense ions (relative intensities above 10%) for samples analyzed after incubation of samples in normal saline. Negative mode.

**Mass**	**0 min**	**35 min**	**70 min**	**315 min**
546.19				572655
547.19				164449,8
548.19				531257,5
549.19				151014,8
550.18				145488,2
570.23				317049,4
571.23				100281,1
572.23				193550
598.49	278404,9	346253,1	370710,9	274658,1
599.50	102247,2	129467	135089,3	
600.51	953755,7	988263,7	1547157	1037186
601.51	372064	381524,2	615425,8	412279,8
602.51	272359	283625,9	514082,1	309823,2
603.51	102692	105252,3	177322	114205,2
628.54	164839,1	129741,7	176705,5	167049,3
654.56			142070,4	
679.51	213332,5	180561,6	213253,3	
722.51			231881,4	
746.51	137822,5		396183,8	
747.52	104447,2		262452,3	
748.53			234069,2	
750.54	145987,5		550561,2	
751.55			246096,3	
762.51	183822,5	115312,7	292874,2	
763.51			129303,4	
764.52			136654,7	
766.54	245981,2	143039,8	688790,4	
767.54	105881,8		309925,5	
768.53	243637,8	216507,3	220399,3	118589,1
772.53			149024,7	
774.54	234697,2	103976,2	611269,1	
775.55	109086,2		294365,4	
776.56			106050,4	
790.54	372874,9	208560,8	813421,7	100922,6
791.54	167562		385141,2	
792.55			112674	
794.55	642902,2	569998,1	395477,8	275557,3
795.55	277641,2	237268,9	177017,1	116057,1
796.54	182489,1	154693,2	103789	
816.53	170255	126263,4		
840.53	133579,5			

865.50	230416,2	105360,5	155457,7	
866.51	113368,5			
885.55	152392,7		190630,1	

Table S3. The description of samples, obtained from each animal. The time from brain removal to sample freezing is provided in the first column. The samples from each hemisphere were taken in random order, except for 12 additional samples from a third animal that was resected from identical brain regions. The time for the first set of samples is not the same as the time required for organ removal, bisecting, and blood removal is slightly varying.

	**Animal 1**				**Animal 2**				**Animal 3**	
	Left hemisphere		Right hemisphere		Left hemisphere		Right hemisphere		Left hemisphere	Right hemisphere
Temp.	+4°C	+4°C	+20°C	+20°C	+20°C	+20°C	+4°C	+4°C	+4°C	+20°C
Storage media	Water	Normal saline	Water	Normal saline	Water	Normal saline	Water	Normal saline	Normal saline	Normal saline
Washing media	Normal saline	Water	Water	Normal saline	Water	Normal saline	Normal saline	Water	Normal saline	Water
2-4 min	2 samples	2 samples	2 samples	2 samples	2 samples	2 samples	2 samples	2 samples	3 samples	3 samples
10 min	2 samples	2 samples	2 samples	2 samples	2 samples	2 samples	2 samples	2 samples	3 samples	3 samples
15 min	2 samples	2 samples	2 samples	2 samples	2 samples	2 samples	2 samples	2 samples	3 samples	3 samples
20 min	2 samples	2 samples	2 samples	2 samples	2 samples	2 samples	2 samples	2 samples	3 samples	3 samples
35 min	2 samples	2 samples	2 samples	2 samples	2 samples	2 samples	2 samples	2 samples	3 samples	3 samples
60 min	2 samples	2 samples	2 samples	2 samples	2 samples	2 samples	2 samples	2 samples	3 samples	3 samples
Comment									Additional 6 samples from each hemisphere were set aside for the immediate analysis.	
